# Talus Position Correlates With Dorsiflexion Range of Motion Following a Lateral Ankle Sprain: A Cross‐Sectional Study

**DOI:** 10.1002/hsr2.70550

**Published:** 2025-03-10

**Authors:** Takeshi Toyooka, Eiki Tsushima, Shiro Sugiura, Yukio Matsushita, Akito Takata, Yasutaka Omori, Yuzuru Okamoto, Satoru Nishikawa

**Affiliations:** ^1^ Department of Rehabilitation Nishikawa Orthopedic Clinic Sakura Japan; ^2^ Graduate School of Health Sciences Hirosaki University Hirosaki Japan; ^3^ Department of Orthopaedic Surgery Graduate School of Medicine Chiba University Chiba Japan; ^4^ Department of Radiology Nishikawa Orthopedic Clinic Sakura Japan; ^5^ Nishikawa Orthopedic Clinic Sakura Japan

**Keywords:** ankle dorsiflexion range of motion, lateral ankle sprain, magnetic resonance imaging, talus alignment

## Abstract

**Background and Aims:**

Following an ankle sprain, clinical examination often reveals ankle dorsiflexion pain, which has been implicated as a risk factor for recurrent ankle sprains; however, the mechanism of ankle dorsiflexion pain has not been explored. Using magnetic resonance imaging (MRI), we evaluated the relationship between the limited dorsiflexion range of motion due to pain and the position of the talus. We investigated whether an anterior talofibular ligament (ATFL) tear affected ankle dorsiflexion.

**Methods:**

We reviewed 36 medical records and MRI images of outpatients diagnosed with a lateral ankle sprain. The data recorded were weight‐bearing lunge test (WBLT), talus alignment, and ATFL tear. Weight‐bearing lunge test differences (WBLTD) between the affected and unaffected sides were calculated. Talus alignment was defined on MRI as the extent of anterior displacement from the posterior lip of the tibia to the nearest articular surface of the talus (distance). Spearman's rank correlation coefficient was used to analyze the relationships between WBLTD and distance. Next, we divided the patients into two groups based on the ATFL tear and compared the WBLTD and distance using the Mann–Whitney *U* test.

**Results:**

The mean and standard deviation for distance and WBLTD were 0.9 ± 0.9 and 3.5 ± 2.8, respectively. Spearman's rank correlation coefficient between distance and WBLTD was 0.48 (*p* = 0.003). There was no significant difference between tear or no‐tear of the ATFL with WBLTD and distance, respectively.

**Conclusion:**

Anterior deviation of the talus positively correlated with dorsiflexion range of motion. Our study highlighted that the talus might have an anterior deviation in the unstressed position. However, the ATFL tear's effects on talar displacement and dorsiflexion angle were unknown.

## 1 Introduction

Various symptoms have been reported in lateral ankle sprains, such as pain during loading, ankle joint instability, and dorsiflexion pain [1, 2]. Among these, dorsiflexion pain significantly impacts daily life, affecting the ability to go downstairs or squat. Studies have also reported that pain during dorsiflexion is not only a typical acute symptom of lateral ankle sprain, but also a risk factor for recurrent ankle sprain [3]. Pain during dorsiflexion can persist as a symptom for a long time [4, 5]. Although triceps tightness is a possible cause of dorsiflexion pain, in clinical practice, dorsiflexion pain after lateral ankle sprains is often reported in the anterior aspect of the ankle joint rather than the triceps muscle [6, 7]. Furthermore, a study comparing triceps stretching and joint mobilization reported that joint mobilization was more effective than triceps stretching [8, 9].

Conversely, synovial fold disorders have been reported as a cause of anterior ankle joint pain and the appearance of osteophytes in the long term [10, 11]. Moreover, fibrous scar tissue may form within the joint following lateral ankle sprain. This formation of scar tissue has been reported to cause impingement in the anterior ankle joint, which results in pain and limited range of motion [12]. Although contact between the distal bundle of the anterior inferior tibiofibular ligament and the talus is normal, studies have reported that this can become pathological and cause anterior impingement in case of ankle joint instability because of injury to the anterior talofibular ligament (ATFL) [13, 14]. Thus, an impingement in the ankle mortise due to ligamentous injury, which interferes with normal joint motion [15], may cause such a condition following a lateral ankle sprain.

Previous studies on talar instability have reported on its anterior and internal rotation displacement in patients with chronic ankle instability [16]. The resulting talus instability within the mortise may cause slow but effective damage to the superficial layer of the ankle cartilage, which is believed to play a major role in resisting the development of osteoarthritis [17, 18, 19]. However, given that these reports focus on the chronic phase, findings in the acute phase remain unknown. Furthermore, the relationship with ankle dorsiflexion range of motion remains unclear. The current study is expected to provide insights on the management of dorsiflexion pain from the acute phase after lateral ankle sprain to clinicians specializing in foot care.

In previous physical therapy reports of the acute phase of lateral ankle sprain, ankle dorsiflexion was found to be potentially limited by the loss of normal posterior glide of the talus in the mortise and by the loss of other accessory motions at the tibiofibular, subtalar, and midtarsal joints [20, 21]. Physical therapy can improve the overall resolution of a lateral ankle sprain, and posterior mobilization of the talus improves ankle dorsiflexion [22, 23, 24, 25]. Denegar and colleagues reported that posterior talar glide was significantly reduced in injured ankles compared with uninjured ankles [26]. These findings suggest a relationship between the position of the talus and ankle dorsiflexion pain; however, there is a lack of research on this relationship. If the talus is already positioned anteriorly because of the sprain, dorsiflexion movement may create increased compression of the anterior joint structures [27], which can cause impingement of the inflamed synovial tissue [28]. Moreover, neurophysiological responses for lateral ankle sprain include increased hypersensitivity of the mechanoreceptors, which can be stimulated by the altered joint's position during movement [29]. Thus, we hypothesized that greater anterior displacement of the talus at rest would result in a more limited ankle dorsiflexion range of motion. The purpose of this study was to use magnetic resonance imaging (MRI) to evaluate the relationship between ankle dorsiflexion range of motion and the amount of anterior talus deviation.

Additionally, the anterior drawer test would come back positive when the ATFL is torn; on the other hand, Caputo and colleagues reported an anterior deviation of the talus even when the ATFL was not torn [30]. However, given that the mentioned study involved patients with chronic ankle instability and was conducted with loading stimuli in the upright position, the position‐related effects of the talus in the unloaded position in the acute phase of the disease remained unclear. Therefore, we investigated whether an ATFL tear affects anterior talar displacement and range of motion. Moreover, determining whether the presence of ATFL tears and talus deviation were associated with dorsiflexion range of motion will facilitate the early management of dorsiflexion pain as a sequela, which has been reported commonly, and prevent subsequent chronic ankle instability.

## 2 Methods

**Figure 1:**
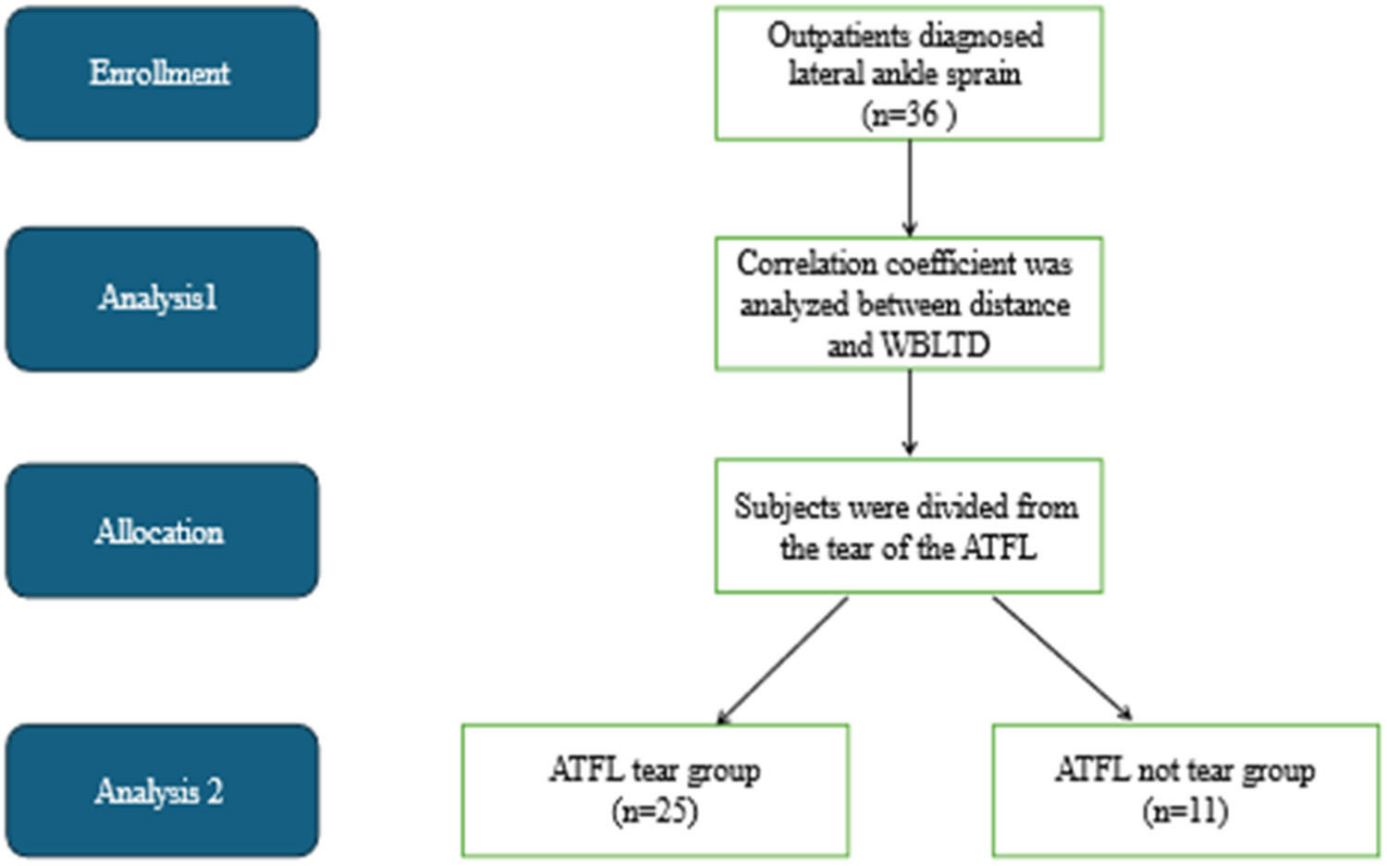
Flow diagram of participant inclusion. WBLTD, the difference between the affected and healthy side of weight‐bearing lunge test; ATFL, anterior talofibular ligament.

From January 2015 to March 2023, outpatients from the author's clinic diagnosed with lateral ankle sprain were assessed. The inclusion criteria were as follows; patients who underwent MRI for diagnostic purposes because of severe pain, those who underwent physical therapy treatment, and those who underwent the WBLT to measure range of motion. The patients include athletes and the general public, whereas the exclusion criteria were as follows: fracture of the foot or ankle, history of ankle surgery, congenital anomaly, or disease duration ≥ 2 weeks (Figure 1). In total, 36 patients were included. The data collected were WBLT, ATFL tear, talus alignment on MRI, disease duration, and the period of MRI inspection from initial diagnosis. The WBLT was one of the measurement methods used to evaluate ankle dorsiflexion ROM. Measuring the WBLT distance has shown excellent interrater and intrarater reliability [31]. The WBLT was measured bilaterally on the affected and healthy side. The difference between the affected and healthy side was defined as the WBLTD. Larger WBLTD values indicate a greater difference in dorsiflexion range of motion between the healthy and affected side. All patients had pain in their affected ankle in the terminal ROM of ankle dorsiflexion.

ATFL tears and talus alignment were evaluated on all patients with a 1.5‐T MRI system (Echelon Vega; Hitachi Medical Corp., Tokyo, Japan). Six images were recorded for each patient with the following MRI sequences: (1) sagittal view of the ankle with a T2‐weighted image (repetition time [TR], 3500 ms; echo time [TE], 104 ms; slice thickness, 4 mm; interslice gap, 1 mm); (2) a T1‐weighted image (TR, 457 ms; TE, 11 ms; slice thickness, 4 mm; interslice gap, 1 mm); (3) a coronal view with fat saturation T2‐weighted image (TR, 2500 ms; TE, 90 ms; slice thickness, 3 mm; interslice gap, 0.3 mm); (4) an axial view with T2‐weighted image (TR, 3200 ms; TE, 90 ms; slice thickness, 4 mm; interslice gap, 0.4 mm); (5) a T1‐weighted image (TR, 436 ms; TE, 10.4 ms; slice thickness, 4 mm; interslice gap, 0.4 mm); and (6) a fat saturation T2‐weighted image (TR, 2800 ms; TE, 90 ms; slice thickness, 4 mm; interslice gap, 0.4 mm). This protocol facilitated diagnosing ATFL tears. ATFL tears were observed on axial view. Although previous studies have reported that ATFL tears can occur in various locations, the probability of diagnosing such tears via MRI is high [32]. Our study focused solely on the presence of a tear to investigate its effect on talus displacement. Therefore, we employed only one clinically experienced orthopedic surgeon to determine the presence of an ATFL tear.

**Figure 2:**
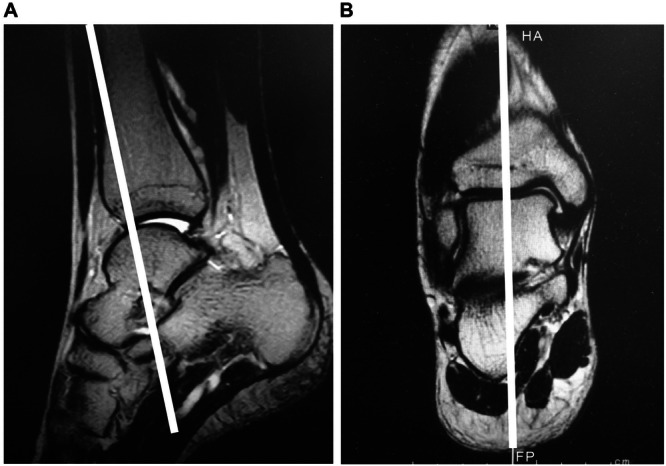
As depicted in the images, we decided on a baseline plane (white line) to evaluate talus alignment. Image on the left side (A) is the sagittal view, and image on the right side (B) is the coronal view.

**Figure 3:**
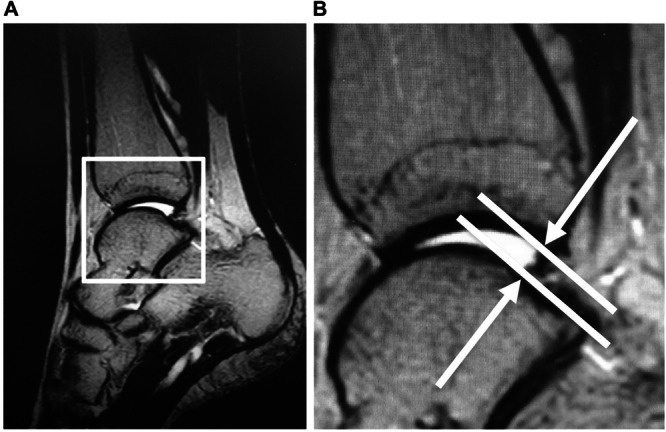
(A) Talus alignment is the extent of anterior displacement (distance) from the posterior lip of the tibia to the nearest articular surface of the talus. (B) An enlarged view of the inset in the panel.

The baseline sagittal view used to observe the talus alignment was defined in two different planes (Figure 2). The axial plane (A) is set parallel to the longitudinal axis of the tibia and runs through the top of the trochlea of the talus on the sagittal plane. The coronal plane (B) was set in the center of the body of the talus. Deviation of the talus in the sagittal view was defined as the extent of anterior displacement from the posterior lip of the tibia to the nearest articular surface of the talus (distance) (Figure 3). This measurement method, which was conceptualized by our group, involves measuring anterior talar displacement using radiographic images as proposed by Hackenbruch et al. [33]. This method showed an ICC (1, 1) and (2, 1) of 0.92(95% confidence interval, 0.70‐0.98) and 0.95(95% confidence interval, 0.79‐0.99), respectively.

**Figure 4:**
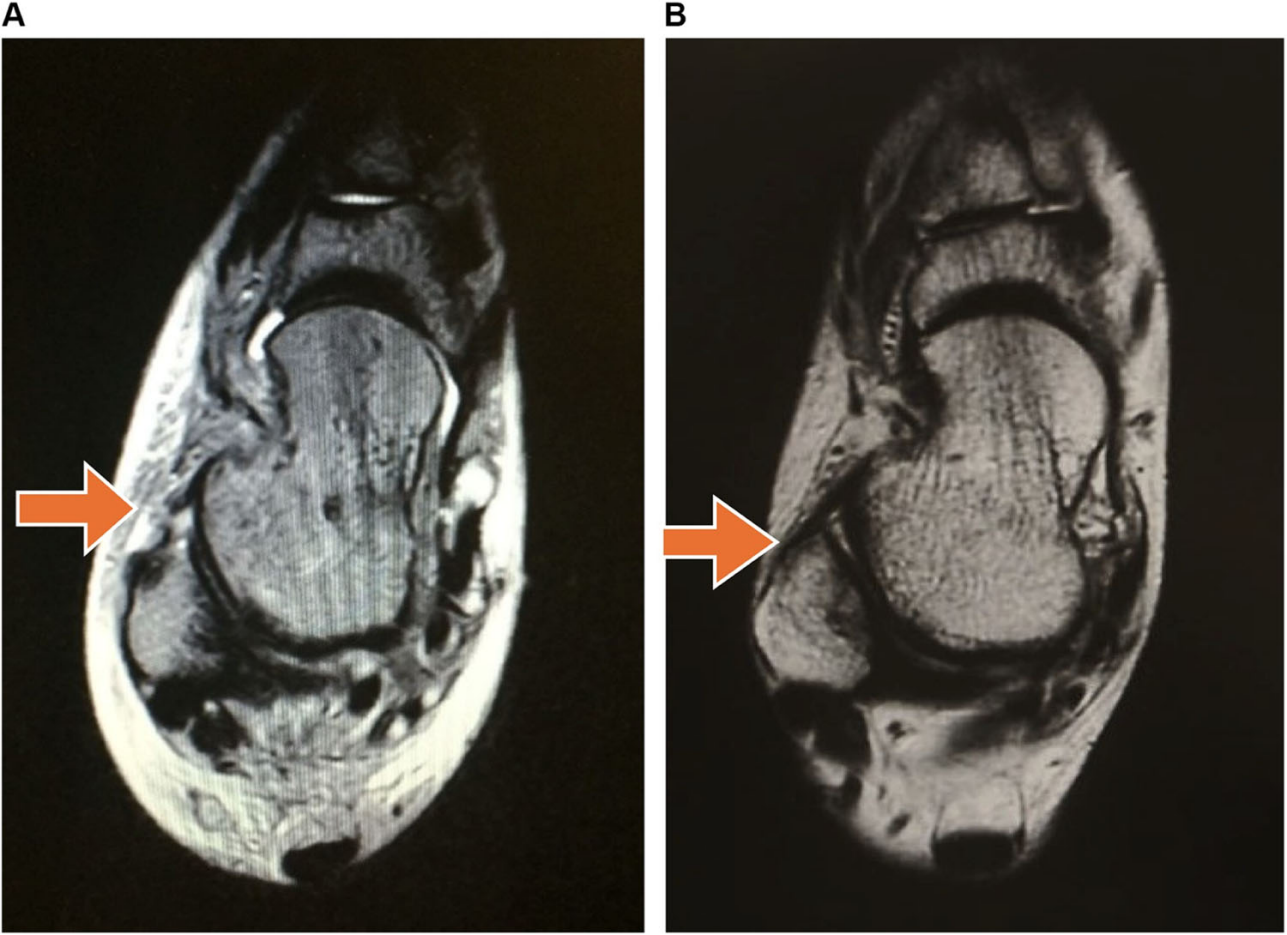
Figure on the left side (A) shows an anterior talofibular ligament (ATFL) tear. The image on the right side (B) shows a normal ATFL near the arrow.

Demographic data and MRI findings are shown in Table 1. They were tested for a normal distribution using Shapiro–Wilk normality tests; however, the data did not follow a normal distribution. First, Spearman's rank correlation coefficient was used to analyze the relationships between distance and WBLTD. Next, we divided patients into two groups (tear group or no‐tear) based on the ATFL tear. Figure 4 shows an MRI image of an ATFL tear and a normal ATFL. The Mann–Whitney U test was used for distance and WBLTD between the tear and no‐tear groups. A *p*‐value < 0.05 was considered significant. Modified R commander 4.2.2 software was used to analyze the data.

**Table 1:** Means and standard deviations of the demographic data and MRI findings.

Characteristic	Mean ± SD
Age, years	17.5 ± 6.7
Sex, male/female	20/16
Body mass index	20.1 ± 3.1
Disease duration, days	1.9 ± 2.6
Period of MRI inspection from initial diagnosis, days	4.3 ± 3.5
Weight‐bearing lunge test, cm, Rt/Lt	8.8 ± 3.7/11.0 ± 2.7
WBLTD^a^, cm	3.5 ± 2.8
Distance, mm	0.9 ± 0.9
Anterior talofibular ligament tear, tear/no‐tear	25/11

^a^ Difference in weight‐bearing lunge test between healthy and affected side.

Our institutional review board (IRB) approved this study (approved No. 2432). Since this is a retrospective study, the IRB determined that informed consent was unnecessary because the surveyed items were related to examinations performed during routine clinical practice. The participants were assured that their results would be kept confidential and that they would not have access to the results of their images.

## 3 Results

**Figure 5:**
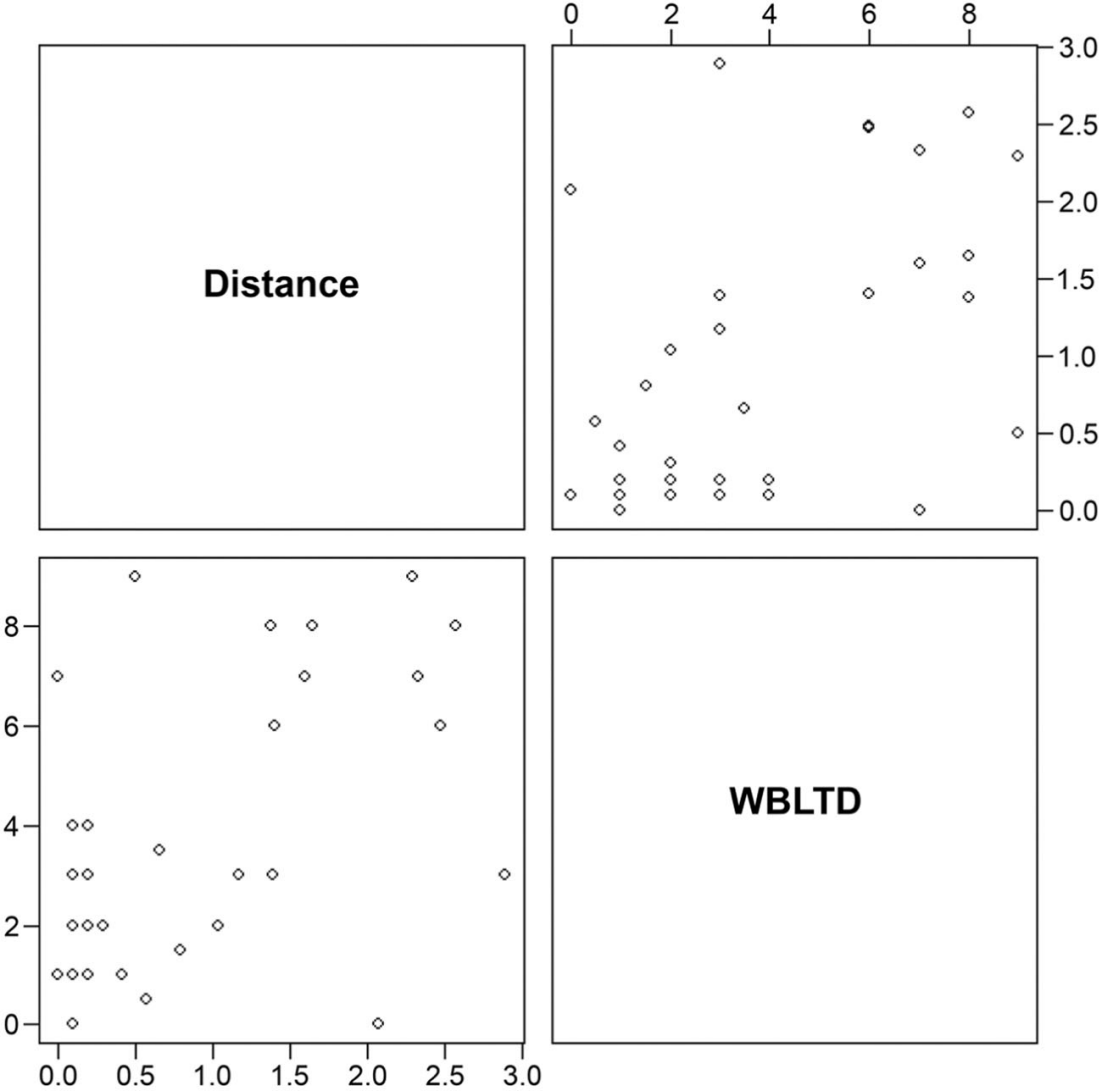
Distance and WBLTD scatter plots.

Spearman's rank correlation coefficient between distance and WBLTD was 0.48 (*p* = 0.003). Figure 5 shows the scatter plots of the distance and WBLTD. The results of the distance and WBLTD when the patients were divided into two groups according to the presence or absence of an ATFL tear are shown in Table 2. There was no significant difference between the tear and the no‐tear group for distance and WBLTD (*p* = 0.69, 0.59, effect size(r): 0.06, 0.09).

**Table 2:** Statistical description (50% = median); upper table: distance; lower table: WBLTD.

	Mean	SD	IQR	0%	25%	50%	75%	100%	data: *n*
Tear	0.89	0.88	1.19	0	0.2	0.5	1.39	2.89	25
No‐tear	0.91	1.03	1.745	0.1	0.1	0.2	1.845	2.57	11
						No significant difference (*p* < 0.05)			

	Mean	SD	IQR	0%	25%	50%	75%	100%	data: *n*
Tear	3.56	2.60	4.00	0.00	2.00	3.00	6.00	9.00	25
No‐tear	3.50	3.43	5.75	0.00	0.75	2.00	6.50	9.00	11
						No significant difference (*p* < 0.05)			

## 4 Discussion

Our results suggest that the greater limitation of the range of motion due to dorsiflexion pain in the standing position was due to the greater anterior variation of the talus bone relative to the tibia. These results align with our expectations, indicating that lateral ankle sprain may cause an anterior shift in the talus during the acute phase after injury. However, we could not determine the effects of an ATFL tear.

In a study on anterior displacement of the talus in healthy ankle joints, Lindstrand and Mortensson reported that the extent of anterior displacement of the talus was < 3 mm when evaluated using radiography [34]. In a three‐dimensional model analysis using fluoroscopic images, Caputo and colleagues reported that the amount of anterior displacement of the talus was 0.2 ± 0.6 mm without anterior drawer stress [30]. Compared with these studies, the present study showed an average anterior displacement of 0.9 mm, which is not exceptionally large compared with values reported for healthy feet in previous studies. Therefore, we believe that the present study failed to determine the effects of ATFL tears on the anterior displacement of the talus.

When the ATFL is torn, the talus is shifted forward, as confirmed by the anterior drawer sign [35]. Karlsson and colleagues reported that the indication for surgery from the anterior drawer sign was 6 mm [36]. Bahr and colleagues reported that with the anterior drawer test when the ATFL was intact, the anterior translation of the talus was ~4 mm. When the ATFL was torn, it was ~6 mm [35]. However, a recent systematic review reported that the anterior drawer test's sensitivity was as low as 54% [37]. The distance was much less than the anterior translation values. Previous studies have evaluated the anterior deviation of the talus under an anterior stressed position (using manual maximum forces or measuring apparatuses) [38]; conversely, our study did not apply anterior drawer stress to the ankle joint of the patients. Our research showed that the distance was < 2 mm in many cases, suggesting that the amount of talus variation was limited within the joint play. Such a small deviation may be difficult to detect on radiographic images. In such cases, MRI would allow clinicians to confirm slight deviations in the talus from various angles. Furthermore, the high ICC obtained in our study makes our approach more reproducible, which we consider more useful than radiography. Clinically, if a gap is found posterior to the talofemoral joint, appropriate physical therapy should be recommended as soon as possible. Furthermore, no significant difference in the dorsiflexion range of motion was observed based on ATFL tear. Hence, we believe that future studies should consider the impact of ATFL tear morphology or other ligament injuries.

There are some limitations in our study. First, the number of patients included was small, and investigation of medical history or numerical assessment of pain intensity during dorsiflexion movements, such as NRS, was not conducted. Second, our study lacked evaluation of the ligaments, such as rupture morphology of the ATFL and lateral ligaments other than the ATFL, and were unable to classify the severity of the injury. Particularly, studies have reported that current MRI techniques are not sensitive enough to differentiate between calcaneofibular ligament injuries [39], highlighting the need for the development of new methods, such as echo evaluation.

It is unclear what mechanism is responsible for the anterior deviation of the talus because no anterior pullout stress is applied during MRI as the cause for anterior deviation. Previous studies have reported that they have altered joint position sense and decreased peroneal muscle strength following lateral ankle ligament injury [40, 41]. If this results in decreased peroneal muscle tone compared with triceps muscle tone, that muscle tension from the triceps may act as a vector that pushes the talus forward because it is located distal to the axis of motion of the talofemoral joint.

Talus deviation can cause future anterior impingement syndromes, such as synovitis or osteophytes because patients with ankle dorsiflexion restriction experience pain in their anterior ankle mortis under weight‐bearing conditions [18]. However, Holland and colleagues reported that posterior mobilization improved ankle dorsiflexion restriction under non‐weight‐bearing conditions but not weight‐bearing conditions [42]. Thus, factors affecting ankle dorsiflexion restriction might differ under weight‐bearing and non‐weight‐bearing conditions. This study evaluated only non‐weight‐bearing conditions. Further research is required to investigate the role of arthrokinematic changes under weight‐bearing conditions. Clinically, if there are patients with ankle dorsiflexion pain, the authors recommend posterior mobilization of the talus as manual therapy and as a physical test of talus deviation because it is thought that ankle dorsiflexion pain is caused by various factors.

## 5 Conclusion

Anterior deviation of the talus was observed on MRI in patients with ankle dorsiflexion pain. Our study highlighted that the talus might have anterior deviation at no stressed and neutral positions and that anterior deviation influences ankle dorsiflexion pain. Moreover, greater anterior displacement of the talus at rest causes more limited ankle dorsiflexion range of motion.

## Author Contributions

**Takeshi Toyooka:** conceptualization (lead), writing – original draft (lead), formal analysis (lead), writing – review and editing (equal). **Eiki Tsushima:** review and editing (equal). **Shiro Sugiura:** software (lead), writing – review and editing (equal). **Yukio Matsushita:** methodology (lead), writing – review and editing (equal). **Akito Takata:** conceptualization (supporting), writing – original draft (supporting), writing – review and editing (equal). **Yasutaka Omori:** conceptualization (supporting), writing – original draft (supporting), writing – review and editing (equal). **Yuzuru Okamoto:** conceptualization (supporting), writing – original draft (supporting), writing – review and editing (equal). **Satoru Nishikawa:** conceptualization (supporting), writing – original draft (supporting), writing – review and editing (equal).

## Conflicts of Interest

The authors declare no conflicts of interest.

## Transparency Statement

The lead author Takeshi Toyooka affirms that this manuscript is an honest, accurate, and transparent account of the study being reported; that no important aspects of the study have been omitted; and that any discrepancies from the study as planned (and, if relevant, registered) have been explained.

## Data Availability

The data that support the findings of this study are available on request from the corresponding author. The data are not publicly available due to privacy or ethical restrictions.
